# Structural Analysis of the Colony-Stimulating Factor 3 Gene of Granulocyte Colony-Stimulating Factor-Producing Urothelial Cancer

**DOI:** 10.7759/cureus.43981

**Published:** 2023-08-23

**Authors:** Yumiko Okuno, Mai Hori, Mami Hattori-Kato, Hiroshi Fukuhara, Akira Nomiya, Koji Mikami, Takumi Takeuchi

**Affiliations:** 1 Department of Urology, Japan Organization of Occupational Health and Safety, Kanto Rosai Hospital, Kawasaki, JPN; 2 Department of Urology, Kyorin University Faculty of Medicine, Tokyo, JPN

**Keywords:** transcription, mutation, csf3, g-csf, urothelial cancer

## Abstract

Background

Granulocyte colony-stimulating factor (G-CSF) is a member of the CSF family of glycoproteins that regulate the proliferation, differentiation, and mobilization of neutrophils. G-CSF-producing malignant cancers have been reported to occur in various organs and are mostly associated with poor clinical prognosis. Here, we analyzed the structure of the *CSF3* gene encoding the G-CSF protein to delineate the mechanism of G-CSF production by the cancer cells.

Methodology

Two cases of G-CSF-producing urothelial cancers and three cases of G-CSF-nonproducing bladder cancers were enrolled for genetic analysis.

Results

In one case of G-CSF-producing bladder cancer, six somatic mutations were detected in the 5’- upstream region of the *CSF3* gene. No somatic mutations in the *CSF3* gene were detected in another case of G-CSF-producing renal pelvic cancer and G-CSF-nonproducing bladder cancers. Copy numbers of the *CSF3* gene were not increased in G-CSF-producing urothelial cancers.

Conclusions

Somatic mutations in the 5’- upstream region of the *CSF3* gene may cause G-CSF protein overproduction.

## Introduction

Granulocyte colony-stimulating factor (G-CSF) is a member of the colony-stimulating factor (CSF) family of glycoproteins that regulate the proliferation, differentiation, and mobilization of neutrophils [1,2]. The human and murine *CSF3* genes encoding G-CSF protein are located on the human q21-22 region of chromosome 17 and murine chromosome 11, respectively, and both genes consist of ﬁve exons and four introns [1-3]. The coding regions of the exons in both genes are highly conserved, with 69% identity. The 300 base pairs upstream from the transcription initiation site are also conserved and have essential promoter sequences. The human *CSF3* gene codes for two different mRNAs that are generated by the alternative use of two 5’ splice donor sequences in intron 2 [2].

Tumor-related leukocytosis is a paraneoplastic syndrome that is rarely observed in patients with nonhematologic malignant tumors [4] and can be caused by the upregulation of granulopoietic cytokines and growth factors, such as G-CSF, granulocyte-macrophage colony-stimulating factor, interleukin (IL)-1, IL-6, and tumor necrosis factor-α [5].

G‑CSF produced by cancer cells also expressing G-CSF receptor can stimulate cancer growth, induce angiogenesis, and promote metastasis by the autocrine signaling pathway [6]. G-CSF-producing malignant cancers have been reported in various organs and are mostly associated with poor clinical prognosis [7].

Innate immune response and subsequent granulopoiesis can be activated by pattern recognition receptors, such as Toll-like receptors (TLRs), expressed on nonhematopoietic cells [8]. For example, TLR4 expression by bladder epithelial cells is mandatory for the initiation of a sufficient immune response against *Escherichia coli* infection in the urinary tract [9]. TLR signaling involves five adaptor proteins, including myeloid differentiation primary-response gene 88 (MyD88), which is the universal adaptor used by all TLRs. Adaptors recruited to Toll/IL-1 receptor (TIR) domains of TLRs initiate signaling [10]. The L265P mutation in MyD88 is a gain-of-function driver mutation [11]. The frequency of mutations in TLR pathways, especially TLR4, is high in patients with esophageal cancer [12].

Here, we observed two cases of G-CSF-producing urothelial cancers (one bladder cancer and one renal pelvic cancer) and analyzed the structures of the *CSF3* gene encoding the G-CSF protein and intracytoplasmic domains of TLRs to delineate the mechanism of G-CSF production by those cancer cells. Specifically for the former, the *CSF3* gene and its upstream sequences in G-CSF-producing urothelial cancer were analyzed by Sanger sequencing, and the copy numbers of the *CSF3* gene were also determined.

## Materials and methods

Cases of G-CSF-producing urothelial cancers

Case 1

A male in his 60s presented with gross hematuria. The diagnosis was invasive bladder cancer. The white blood cell (WBC) count was 17,300 at diagnosis. Transurethral resection of the bladder tumor (TURBT) was performed, and the pathology was urothelial cancer with muscle invasion. In addition, immunohistochemistry showed G-CSF expression in the cytoplasm of tumor cells. Radical cystoprostatectomy with ileal conduit was performed, and the pathology revealed no remaining tumor, with pathological stage pT0N0M0. Nevertheless, multiple lung metastases occurred after one month. Gemcitabine plus cisplatin chemotherapy was not effective, and the patient died due to cancer 3.4 months following the diagnosis of metastases. At death, the WBC count was 139,500.

Case 2

A male in his 60s presented with gross hematuria. The diagnosis was a left renal pelvic tumor with multiple lung metastases. The WBC count was 20,000. Administration of two courses of gemcitabine plus cisplatin chemotherapy appeared to stabilize the original and metastatic tumors. Then, a left nephroureterectomy was performed. Before the operation, the WBC was 25,700, and the serum level of G-CSF was 172 pg/mL (normally ≤39). The pathology of the surgical specimen was urothelial cancer of the left renal pelvis, with pathological stage pT2N0M1. Immunohistochemistry showed G-CSF expression in the cytoplasm of tumor cells. Six weeks after the nephroureterectomy, lung metastases increased. Pembrolizumab, an anti-programmed death receptor-1 (PD-1) antibody, was administered, but it was not effective. At cancer death 6.5 months following left nephroureterectomy, the WBC count was 94,400.

Case 3

A female in her 60s presented with gross hematuria. The diagnosis was large bladder cancer. The WBC count was 25,100 and the serum level of G-CSF was 409 pg/mL at diagnosis. TURBT was performed, and the pathology was urothelial cancer with submucosal invasion. Immunohistochemistry showed G-CSF expression in the cytoplasm of tumor cells. After two months, a bladder tumor measuring 7 cm in diameter rapidly relapsed and transurethral tumor resection was incomplete. Then, an anti-PD-1 antibody therapy using pembrolizumab was introduced. A temporary increase in the bladder tumor and worsening of the bilateral hydronephrosis were observed, but at the end of four courses, the WBC was normalized to 7,800, and the disappearance of the bladder tumor was confirmed by a CT scan and cystoscopy. Eight courses of pembrolizumab were administered in total, and complete remission was achieved with no bladder tumor recurrence up to 20 months after starting the therapy. In Case 3, the bladder tumor specimen for genetic analysis could not be collected.

Control 1

A male in his 80s underwent TURBT for a bladder tumor. TURBT pathology report showed urothelial carcinoma, pT1, G3>G2. Preoperative WBC was 5,800. Bacille Calmette-Guerin bladder instillation therapy was performed after TURBT. One and two years later, the patient developed a recurrence in the bladder and underwent TURBTs again. There has been no recurrence since then.

Control 2

A male in his 60s underwent TURBT for a bladder tumor. TURBT pathology report showed urothelial carcinoma, pTa, G2>G3. Preoperative WBC was 6,400. One and two years later, the patient developed a recurrence in the bladder and underwent TURBTs again. Four years after the initial treatment, the patient died of colorectal cancer.

Control 3

A male in his 80s underwent TURBT for a bladder tumor. TURBT pathology report showed urothelial carcinoma, pT1, G3>G2. Preoperative WBC was 5,300. One year after TURBT, the patient died of pneumonia.

Two cases of G-CSF-producing urothelial cancers (Cases 1 and 2) and three cases of G-CSF-nonproducing bladder cancers (Controls 1-3) were enrolled for genetic analysis. Genomic DNAs were extracted and purified from surgical specimens kept at −70°C until use, as well as whole blood samples collected in ethylenediaminetetraacetic acid-containing tubes using kits (DNA Extractor® WB Kit and DNA Extractor® TIS Kit, Fujifilm Wako Pure Chemical Corporation, Osaka, Japan), following the manufacturer’s instructions. Bladder and renal pelvis cancers share a similarity in that both are included in the category of urothelial carcinoma.

Immunohistochemistry of G-CSF

Paraffin-embedded sections of surgical specimens were immunohistochemically stained with anti-human mouse monoclonal G-CSF antibody (Santa Cruz Biotechnology, Dallas, TX, Cat.no. sc-53292), following the manufacturer’s instruction.

Sequencing of the *CSF3* gene

For genomic DNAs, the *CSF3* gene was amplified in three overlapping fragments (Fragments 1, 2, and 3) encompassing the whole gene using sense/antisense pairs of primers. Polymerase chain reactions (PCRs) were performed using a thermal cycler (Thermal Cycler Wako WK-0518, Wako, Osaka, Japan) in volumes of 50 μL with 0.3 μM of sense and antisense primers, one unit of KOD Plus ver.2 polymerase (Toyobo, Osaka, Japan), 4% dimethyl sulfoxide (DMSO), and the buffer supplied with the enzyme as follows: 94°C for two minutes, 36 cycles (98°C for 10 seconds for denaturing, 68°C for two minutes for annealing and extension) of two-step PCR. Amplified PCR products (10 μL) were resolved by electrophoresis in 1% agarose gel. The primers used for amplification were 5’-tcgagaccagcctgaccaccaacatgg-3’/5’-ctgggccaagacactcacccatcagct-3’ for Fragment 1 (2,005 bps), 5’-gggcaaggcgacgtcaaaggaggatca-3’/5’-cccgaggccacccagaaaaacaggaga-3’ for Fragment 2 (2,345 bps), and 5’-ccaggcctctgtgtccttccctgcatt-3’/5’-ggaaagcagcttcccttccttggagcc-3’ for Fragment 3 (1,867 bps). PCR products were directly Sanger sequenced with PCR primers and additional sequencing primers; 5’-atcacgaggtcaggagatcgtgac-3’ and 5’-aactctccggaggctgcctgtctg-3’ for Fragment 1; 5’-gcttcctgctcaagtgcttagagc-3’ for Fragment 2; 5’-cttgagtccagctggtgcctggcc-3’ and 5’-gtcacattgtaactgaacttcagg-3’ for Fragment 3. The analyzed sequences were compared with a reference sequence obtained from NCBI Reference Sequence: NC_000017.11.

Copy number analysis of the *CSF3* gene

Assays were designed according to the guidelines from Bio-Rad Laboratories. Copy Number Determination Assay kits for CSF3 and AP3B1 were purchased from Bio-Rad Laboratories (CA, USA).

A 22-μL mixture containing 1.1 μL of Copy Number Determination Assay: CSF3, 1.1 μL of Copy Number Determination Assay: AP3B1, 11 μL of ddPCRTM Supermix for Probes (no dUTP) (Bio-Rad), 0.5 μL of HaeIII enzyme (New England Biolabs), and 20 ng of DNA resuspended in dH_2_O was incubated using a T100 Thermal Cycler for restriction enzyme treatment, followed by droplet generation using Automated Droplet Generator Oil for Probes (Bio-Rad).

PCR with the droplets was performed using a Veriti® Thermal Cycler (Thermo Fisher Scientific) with the following cycling parameters: 10 minutes at 95°C (one cycle), 30 seconds of denaturation at 94°C and one minute of annealing and extension at 60°C (40 cycles), 10 minutes at 98°C, and hold at 4°C. All steps had a ramp rate of 2°C/second. Fluorescence signals of the droplets were measured using a QX200TM Droplet Reader (Bio-Rad). Fluorescent data from each well were analyzed using QuantaSoftTM Analysis Pro Software, calculating copy numbers based on the Poisson distribution. Copy numbers of the *CSF3* gene were expressed assuming that the copy number of the *AP3B1* gene in the corresponding genome is two. Copy number analysis of the *CSF3* gene was performed by Riken Genesis Co., Ltd. (Tokyo, Japan).

Sequencing of TLR2 TIR, TLR4 TIR, and *MyD88* gene

The PCR and gel electrophoresis conditions were similar to those in the *CSF3* gene, except the annealing and extension time was 30 seconds. Primers for amplification were 5’-ccgtttccatggcctgtggtatatgaa-3’/5’-ctaggactttatcgcagctctcagat-3’ for TLR2 TIR (526 bps), 5’-acctgatgcttcttgctggctgcataaag-3’/5’-tcagatagatgttgcttcctgccaattgca-3’ for TLR4 TIR (551 bps), and 5’-gggatatgctgaactaagttgccac-3’/5’-gacgtgtctgtgaagttggcatctc-3’ for *MyD88 *(726 bps). The PCR products were directly sequenced with PCR primers.

## Results

Immunohistochemistry of G-CSF

**Figure 1:**
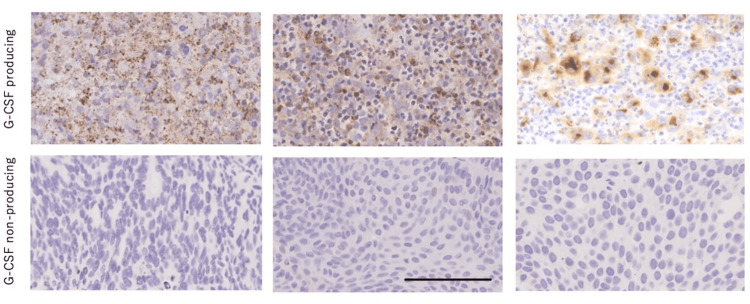
Immunohistochemistry of G-CSF in urothelial cancers. Upper: G-CSF-producing urothelial cancers for Cases 1, 2, and 3 from the left. Lower: G-CSF-nonproducing bladder cancers for Controls 1, 2, and 3 from the left. Bar = 50 μm. The scale bar entered applies to all photos. G-CSF = granulocyte colony-stimulating factor

By immunohistochemistry, G-CSF-producing urothelial cancers showed cytoplasmic G-CSF protein expression, whereas G-CSF-nonproducing bladder cancers did not (Figure 1).

Sequencing of *CSF3* gene

**Figure 2:**
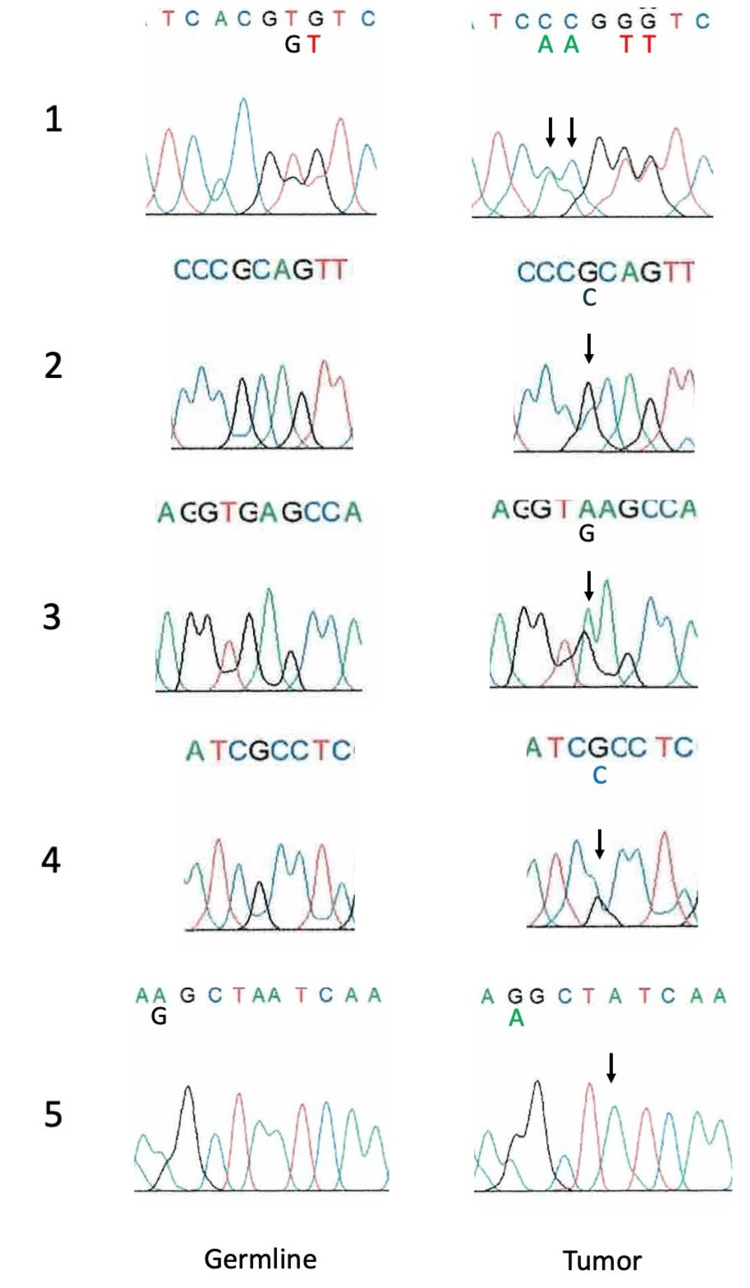
Somatic mutations in the 5’-upstream region of a G-CSF-producing bladder cancer case (Case 1). 1. c.-878A>C (heterozygous) and c.-877C>A (heterozygous, same as rs565504795), 2. c.-863G>C (heterozygous), 3. c.-800G>A (heterozygous), 4. c.-791G>C (heterozygous), 5. c.-667delA (homozygous), See also Table 1. G-CSF = granulocyte colony-stimulating factor

**Figure 3:**
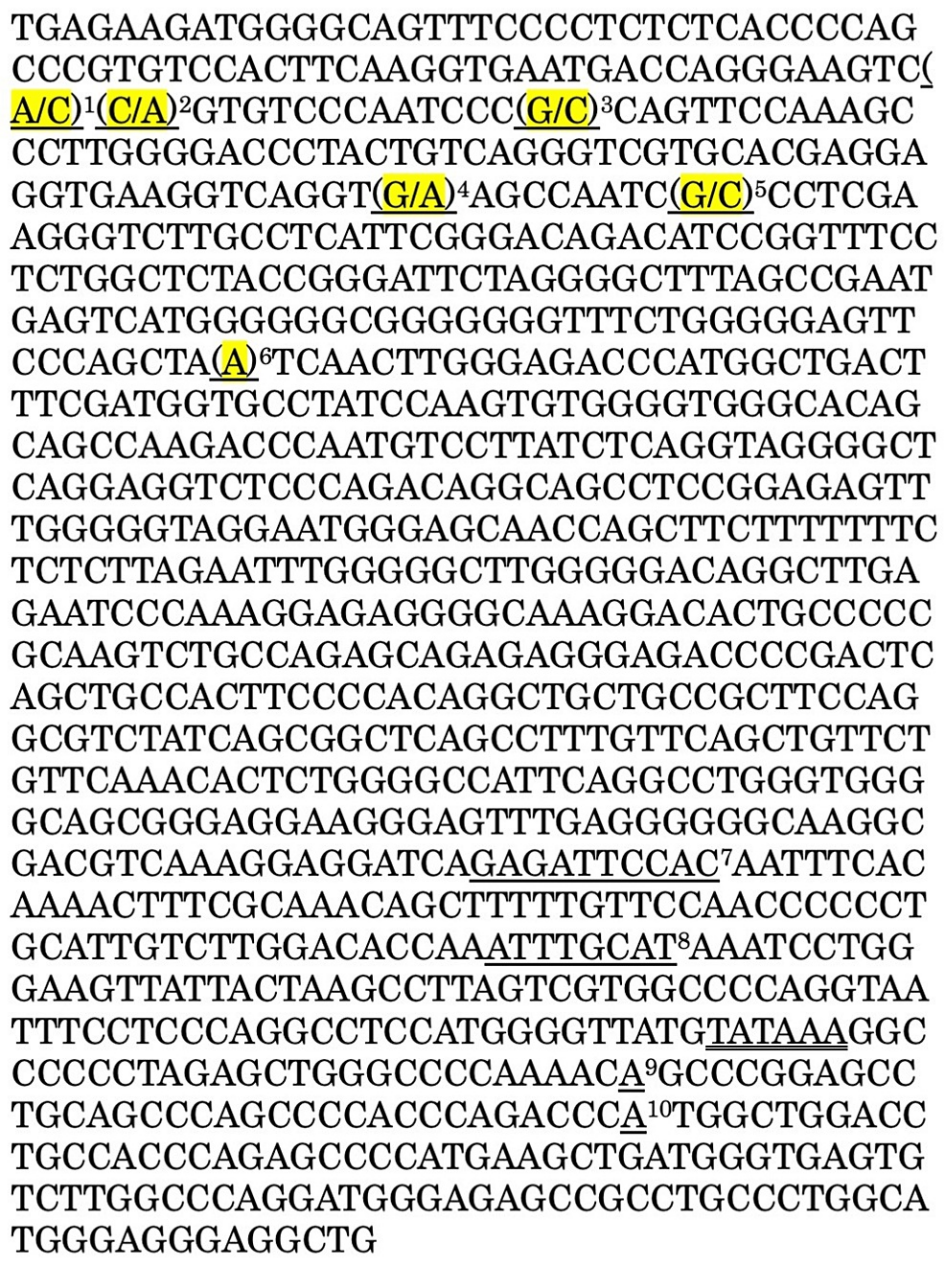
5’-upstream region sequence of the CSF3 gene in G-CSF-producing bladder cancer (Case 1). 1-6 (highlighted) indicate somatic mutations. 1. c.-878A>C (heterozygous), 2. c.-877C>A (heterozygous, same as rs565504795), 3. c.-863G>C (heterozygous), 4. c.-800G>A (heterozygous), 5. c.-791G>C (heterozygous), 6. c.-667delA (homozygous), 7. GPE-1: CSF box, decanucleotide, 192bp upstream from the transcription initiation site, 8. GPE-2: OTF binding site, octamer sequence, 116 bp upstream from the transcription initiation site, 9. Transcription start site, 10. Translation start site, double underline: TATA box, simple single nucleotide polymorphisms are not described here. See also Figure 2 and reference [13]. G-CSF = granulocyte colony-stimulating factor

In the case of G-CSF-producing bladder cancer (Case 1), six somatic mutations were detected in the 5’-upstream region of the *CSF3* gene (Figures 2, 3), but no somatic mutations were found in other regions, including exons. No somatic mutations in the *CSF3* gene were detected in the other case of GSCF-producing renal pelvic cancer (Case 2) or three cases of G-CSF-nonproducing bladder cancer.

Copy number analysis of the *CSF3 *gene

As shown in Table 1, copy numbers of the *CSF3* gene were not increased in G-CSF-producing urothelial cancers.

**Table 1:** Copy numbers of the CSF3 gene in G-CSF-producing urothelial cancers. G-CSF = granulocyte colony-stimulating factor

Cases	Copy number of the G-CSF gene
G-CSF-producing bladder cancer (Case 1)	2.1
G-CSF-producing renal pelvic cancer (Case 2)	1.5
G-CSF-nonproducing bladder cancer (Control 1)	3.3
G-CSF-nonproducing bladder cancer (Control 2)	2.0
G-CSF-nonproducing bladder cancer (Control 3)	1.2

Sequencing of the TLR2 TIR, TLR4 TIR, and *MyD88* gene

There were no variations in the DNA sequences of the *MyD88 *gene or the TIRs of TLR2 and TLR4 compared with reference sequences in all patients.

## Discussion

**Figure 4:**
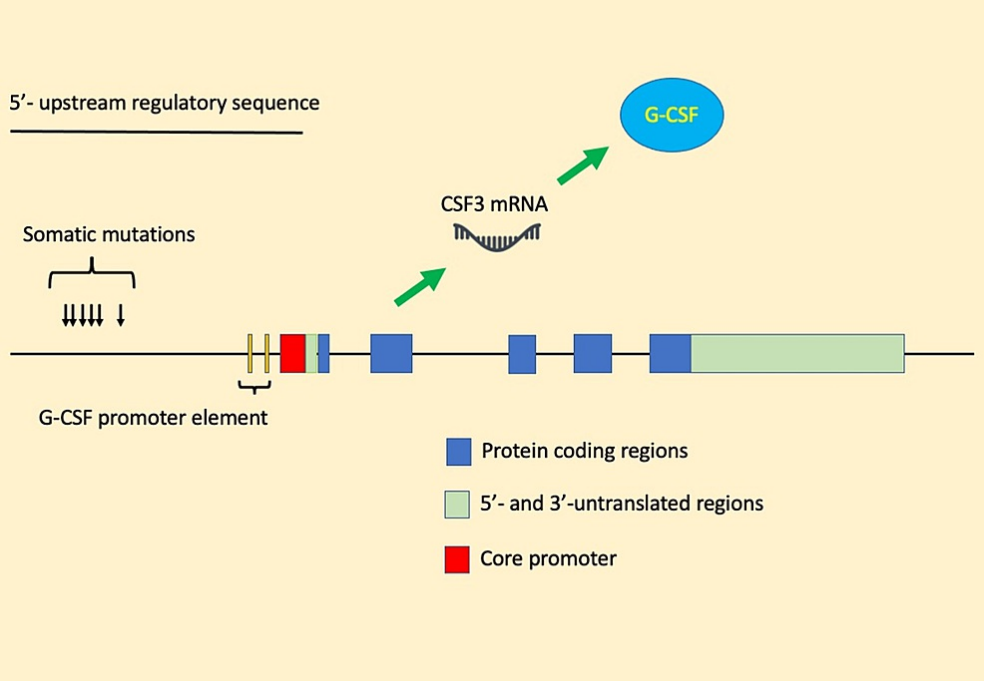
Graphical summary. G-CSF = granulocyte colony-stimulating factor

It is not well understood why G-CSF-producing tumors overproduce the G-CSF protein. In this study, we detected six somatic mutations in the 5’-upstream region of the *CSF3* gene in G-CSF-producing bladder cancer. These mutations may affect the promoter activity of the *CSF3* gene and lead to G-CSF protein overproduction (Figure 4). However, if this is the case, it is not possible to determine which somatic mutations contribute to G-CSF overproduction in this study.

Nishizawa et al. analyzed the promoter structure and function of the mouse *CSF3* gene and identified three well-conserved transcriptional regulatory sites in promoter regions 192, 116, and 87 bp upstream of the transcription start site: G-CSF promoter element (GPE)-1, GPE-2, and GPE-3, respectively. Mutations introduced further upstream of GPE-1 (CSF box) did not affect transcription [13]. The six somatic mutations detected in this study were all upstream of the decanucleotide corresponding to GPE-1 (Table 1), but because of species differences between humans and mice and the multiplicity of somatic mutations, the possibility that they alter the promoter activity of the *CSF3* gene cannot be excluded.

In general, an increased gene copy number may be responsible for increased protein production. However, the copy number of the *CSF3* gene was not increased in the two G-CSF-producing urothelial cancers, so the increased copy number of the *CSF3* gene was not considered a cause of G-CSF overproduction in the cases examined herein.

Granulopoiesis can also be triggered by signaling from TLRs. In this study, we examined the sequences of the TLR2 and TLR4 TIR domains, as well as MyD88, the major adaptor to TIR, in G-CFS-producing urothelial cancer tissues, but found no somatic mutations. Thus, TLRs may not be involved in the overproduction of G-CSF by tumor cells.

It is undeniable that somatic mutations in the 5’-upstream region of the *CSF3* gene caused the overproduction of G-CSF protein in the G-CSF-producing bladder cancer examined in this study. However, in the case of G-CSF-producing renal pelvic cancer (Case 2), neither the somatic mutation in the *CSF3* gene, the increased *CSF3* gene copy number, nor the abnormal signaling from TLRs can explain the overproduction of G-CSF protein. In this case, genomic structures other than the *CSF3* gene, such as those adjacent to the *CSF3* gene, aberrated methylation of the *CSF3 *gene, or factors other than TLR signaling that can induce granulopoiesis, may have had an effect. For example, GeneHancer [14] has shown that an enhancer (GH17J040014) located 3.8 kb away from the transcription start site is involved in the transcription of the *CSF3* gene.

As seen in Cases 1 and 2, the prognosis of G-CSF-producing urothelial cancer is poor. While cisplatin-based chemotherapy has been widely used for advanced and metastatic urothelial cancer, effective treatment for chemotherapy-resistant urothelial cancer is almost nonexistent. Recently, immunotherapy using anti-PD-1 and anti-programmed cell death ligand 1 (PD-L1) antibodies has been introduced and shown some efficacy [15-17]. As shown in this study, a rapidly growing G-CSF-producing bladder cancer that was difficult to control endoscopically went into complete remission with anti-PD-1 antibody therapy. The results suggest that immunotherapy is effective in some cases of difficult-to-treat G-CSF-producing urothelial cancer. Additionally, a previous case report showed that pembrolizumab produced complete remission in metastatic G-CSF-producing bladder cancer [18], and another report demonstrated that G-CSF-producing lung cancers express high levels of PD-L1, which supports our interpretation [19].

A limitation of this study is that it presents only one possible cause for the formation of G-CSF-producing tumors. Various other causes of G-CSF-producing tumors should be investigated in multiple cases, with the hope of elucidating therapies specific to G-CSF-producing tumors.

## Conclusions

In one case of G-CSF-producing bladder cancer, six somatic mutations were detected in the 5’- upstream region of the *CSF3 *gene, suggesting that they cause G-CSF protein overproduction. An increased copy number of the *CSF3* gene was not observed in the two cases of G-CSF-producing tumors studied.
